# Microbial-Driven Immunological Memory and Its Potential Role in Microbiome Editing for the Prevention of Colorectal Cancer

**DOI:** 10.3389/fcimb.2021.752304

**Published:** 2021-11-12

**Authors:** Laure Campillo-Gimenez, David Rios-Covian, Jesus Rivera-Nieves, Hiroshi Kiyono, Hiutung Chu, Peter B. Ernst

**Affiliations:** ^1^ Department of Pathology, University of California San Diego, San Diego, CA, United States; ^2^ Department of Medicine, Division of Gastroenterology, University of California San Diego, San Diego, CA, United States; ^3^ San Diego Veterans Affairs (VA) Medical Center, San Diego, CA, United States; ^4^ CU-UCSD, Center for Mucosal Immunology, Allergy and Vaccine Development, University of California San Diego, San Diego, CA, United States; ^5^ Future Medicine Education and Research Organization, Chiba University, Chiba, Japan; ^6^ Division of Comparative Pathology and Medicine, University of California San Diego, San Diego, CA, United States

**Keywords:** microbiome, IBD, CRC, T/B cell repertoire, immune memory, microbiome-editing

## Abstract

Over the last several years, many advances have been made in understanding the role of bacteria in the pathogenesis of gastrointestinal cancers. Beginning with *Helicobacter pylori* being recognized as the first bacterial carcinogen and the causative agent of most gastric cancers, more recent studies have examined the role of enteric microbes in colorectal cancer. In the digestive tract, these communities are numerous and have a complex interrelationship with local immune/inflammatory responses that impact the health of the host. As modifying the microbiome in the stomach has decreased the risk of gastric cancer, modifying the distal microbiome may decrease the risk of colorectal cancers. To date, very few studies have considered the notion that mucosal lymphocyte-dependent immune memory may confound attempts to change the microbial components in these communities. The goal of this review is to consider some of the factors impacting host-microbial interactions that affect colorectal cancer and raise questions about how immune memory responses to the local microbial consortium affect any attempt to modify the composition of the intestinal microbiome.

## Introduction

Bacteria – specifically *H. pylori* - have been implicated in cancers of the gastrointestinal tract (Marshall and Warren, 1983; Sears and Garrett, 2014) and this species has now been described as a Group 1 carcinogen by the WHO (Forman, 1996; Fu and Xie, 2019; Cho et al., 2021). Changes in the gastric microbiome occur in association with *H. pylori* infection but their impact on colonization, bacterial burden, gene expression and susceptibility to adverse outcomes remain to be determined (Brawner et al., 2014). Removing the infection with anti-microbial protocols decreases the risk of gastric cancer significantly (Crowe, 2019).

As Correa (Correa et al., 1976) predicted, an important factor contributing to cancer caused by *H. pylori* is the oxidative stress associated with chronic inflammation as it increases oxidative DNA damage and mutations leading to malignant transformation (Ernst et al., 2006; Hoeijmakers, 2009; Bhattacharyya et al., 2014; Chumduri et al., 2016). Other causes of chronic inflammation in the digestive tract are associated with cancer including: duodenal tumors with unmanaged or refractory celiac disease (Sharaiha et al., 2012); and inflammatory bowel diseases (IBD) leading to colorectal cancer (CRC) (Irrazabal et al., 2020; Zouggar et al., 2020). IBD has been associated with dysbiosis (Elson and Cong, 2012; Winter et al., 2013; Schaubeck et al., 2015; Honda and Littman, 2016; Dixit et al., 2021) that favors inflammation and enhances the presence of genotoxic strains of bacteria that impart DNA damage (Arthur et al., 2012). Thus, the host response is inextricably linked to the host’s microbiome with both factors contributing to health or disease, including CRC. The purpose of this review is to propose the hypothesis that immunological memory, conferred by the antigen-specific T helper (Th) and B cell responses in health or disease, “shapes” the composition of the microbiological communities. Furthermore, in trying to alter the microbiome for therapeutic benefit, these memory responses would favor the return of the dysbiosis.

## Unique Aspects of Immunological Memory in the Digestive Tract

Antigen-presenting cells (especially dendritic cells) recognize infections, process, and present microbial antigens to Th cells leading to a pool of antigen-specific memory T and B lymphocytes with the capacity to rapidly expand an immune response to a previously encountered stimulus. While innate cells contribute to “trained immunity” (Bekkering et al., 2021), adaptive immunity is the focus for this discussion on immunological memory. Evidence for memory responses in the gut include the presence of antigen-specific IgA B cell responses after immunization with cholera toxin in mice (Lange et al., 1980; Lycke and Holmgren, 1989) or humans (Quiding et al., 1991). Memory B cell responses have also been reported for rotavirus (Moser et al., 1998). These studies indicate that secondary responses are accelerated and one can reasonably assume that they occur to most of the persistent microbes throughout the digestive tract.

The immunological challenge in the digestive tract requires a measured response to the enormous antigenic burden such that it will not induce inflammation and damage the epithelial surface. Evidence for IgA’s protective role is found in studies showing that the absence of IgA is associated with inflammatory disease in the gut (Nagaishi et al., 2021). IgA is a preferred effector as it can block adherence or neutralize toxins without activation of complement (Marshall et al., 1989; Cerutti and Rescigno, 2008). Secretory IgA binds microbes and also modulates bacterial gene expression and their colonization (Donaldson et al., 2018; Sterlin et al., 2020; Guo et al., 2021). In this manner, IgA limits acute infections with pathogens and establishes an equilibrium with other organisms that are beneficial, or pose no immediate threat. Thus “antigen-specific” IgA establishes a relatively stable, homeostatic environment shared by the host and local microbes. To be clear, not all host responses that enable a microbial species to colonize will be desirable as illustrated by *H. pylori*. This organism persists throughout life, but being a pathobiont, it can become pathogenic. Thus, homeostatic memory may enable organisms of varying virulence to persist. This concept is complicated by the fact that a single clone of IgA can bind multiple species of bacteria (Bunker et al., 2017; Pabst and Slack, 2020; Sterlin et al., 2020).

In contrast to the healthy gut, IgG is the predominant mucosal isotype in gastritis associated with *H. pylori¸* celiac disease and IBD (Brandtzaeg et al., 1986). IgG responses are coupled with the accumulation of activated complement in the inflamed stomach (Berstad et al., 2001) and colon (Berstad et al., 1997). The persistent change in isotype may reflect an infective attempt to clear the organism driving the response. In humans with Crohn’s disease, intestinal bacteria are coated with IgA and IgG whereas in ulcerative colitis, just IgG (Rengarajan et al., 2020). Inoculation of susceptible mice with antibody-coated microbes induces disease (Palm et al., 2014). Thus, IgA can respond to bacteria that promote health or disease and as such, pre-existing IgA responses may select for the species of bacteria that induced them and favor the return of the previous health condition.

Th cell responses are the major driving force for enabling the preferred B cell response. These include control of isotype switch, for example to IgA, as well as the expansion of antigen-specific B cell clones. Certain species of bacteria shape the B cell repertoire (Li et al., 2020), favor the induction of IgA (Yang et al., 2020), including some T cell-independent responses (Pabst and Slack, 2020), and will impact homeostasis accordingly. Memory T cell responses in the mucosa have been described and include Th cells (Offit et al., 1992) as well as cytotoxic T lymphocytes (CTL) (Offit et al., 1991; Cheroutre and Madakamutil, 2004) recognizing rotavirus. Another example of a memory response would be the acquired “oral tolerance” that develops to antigens persistently administered orally (Tokuhara et al., 2019). While much remains to be learned about how these populations contribute to health, compelling evidence suggests intestinal regulatory T cells (Treg) display a classic memory T cell phenotype (Guo et al., 2008) and favor a homeostatic interaction with persistent colonization by specific microbes.

Another property of tissue-resident memory Th cells is their dependence on adhesion molecules (i.e. α4β7 integrin) (Lawrance, 2012; Ley et al., 2016), chemokines (Dixon et al., 2016; Perez-Jeldres et al., 2019) and chemokine receptors (i.e. CCL25/CCR9) that control the trafficking of memory lymphocytes among mucosal tissues (Wong et al., 2016; Fu et al., 2019). In this manner, gut memory “does not fade” (Szilagyi et al., 2017) and ensures memory lymphocytes encounter reinfections and expedite secondary responses. Evidence that these interactions contribute to host-microbial interactions is found when immunotherapies targeting these interactions in IBD (Lawrance, 2012; Perez-Jeldres et al., 2019) and refractory celiac disease remove the inflammatory cells and decrease inflammation. Thus, in disease, there can be a persistent antigen-host response dialogue and some of these triggers will include microbes.

## The Contribution of Microbial Colonization to Immunological Memory

**Figure 1:**
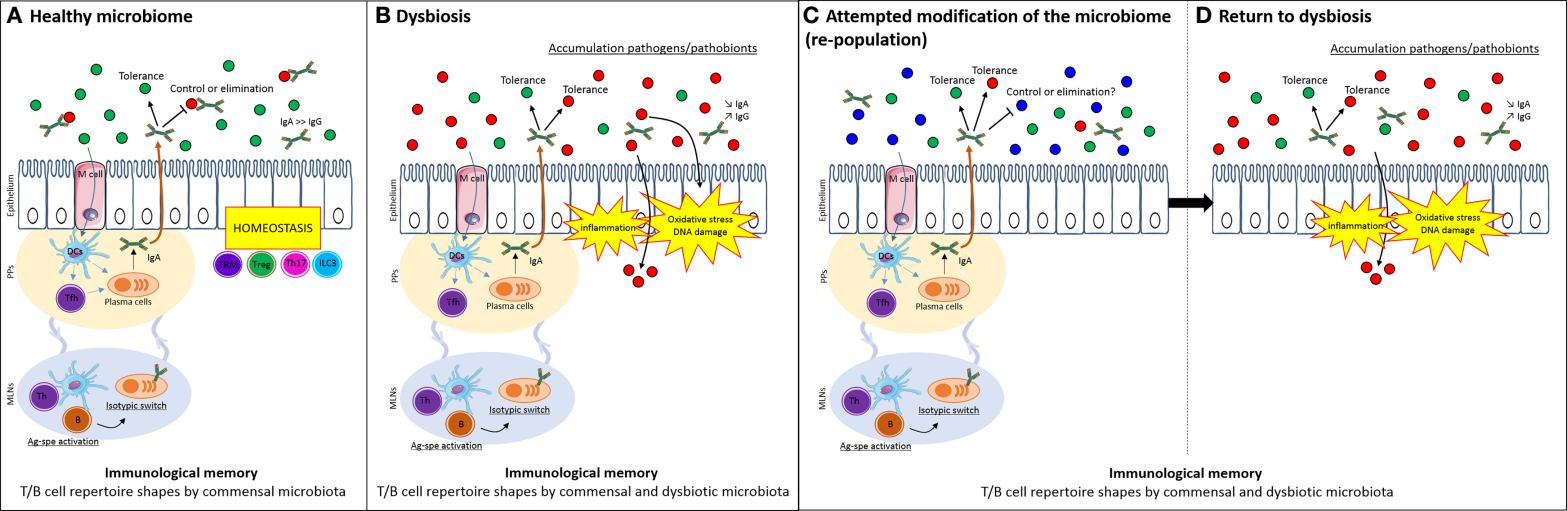
Immunological memory selects for familiar microbiota and limits attempt to modify microbiome. **(A)** The host can have a balanced homeostatic relationship that inhibits (through any innate or adaptive mechanism) the growth of deleterious bacteria (in particular, those that cause acute disease, red dots) while co-existing with symbiotic species (green dot) or even species that pose no immediate risk but may contribute in the future to chronic disease such as IBD or CRC (e.g. a pathobiont, red dots). Antigen-specific activation of T and B cells imparts the immunological memory to the existing microbiome. **(B)** In dysbiosis, microbes favoring inflammatory responses and carcinogenesis exist in the presence of host responses that are also antigen-specific. These responses have adapted to favor the persistence of the dysbiotic microbial population. **(C)** When attempts are made to modulate the microbiota – without altering the immunological memory - existing host responses from the dysbiotic state persist and **(D)** favor the re-selection of the same community, provided through the therapeutic inoculum (such as a fecal transplant) or additional encounters in the host’s normal environment, e.g. microbes shared from family members, dietary ingredients and so on. DCs, dendritic cells; Tfh, follicular T cells; Th, helper T cells; T_RM_, tissue-resident memory T cells; Treg, regulatory T cells; B, B cells; IgA, immunoglobulin A; IgG, immunoglobulin G; Ag-spe activation, antigen-specific activation; PPs, Peyer’s patches; MLNs, mesenteric lymph nodes.

Since Pasteur, host-microbial relationships have been acknowledged to reflect their respective co-evolution. Under germ-free conditions, there is a paucity of immune and inflammatory cells in the gut (Hapfelmeier et al., 2010). As colonization progresses, inductive sites, such as Peyer’s patches and draining lymph nodes, enlarge and seed lymphoblasts that traffic throughout the gut and other mucosal tissues (McDermott and Bienenstock, 1979; Guy-gr et al., 1984; McDermott et al., 1985; Kurashima and Kiyono, 2017; Zhao and Elson, 2018; Chen et al., 2020). If microbiota are necessary to activate intestinal immune responses, it is reasonable to assume that they will favor immunological memory and homeostasis (Marshall et al., 1989). Further, this relationship may have to be “broken” to enable the colonization of the gut with new microbial species administered for their health benefit (**Figure 1A**).

T-cell and B-cell receptors (respectively TCR and BCR) recognize antigens that reflect prior exposure to microbes throughout life (Chu et al., 2019; Kreuk et al., 2019; Chen et al., 2020; New et al., 2020). Regionalization of the repertoire is reflected in intestinal intraepithelial lymphocytes as they display a higher oligoclonality compared to peripheral lymphocytes (Regnault et al., 1995). These repertoires comprise some of the evidence for the co-evolution of host responses and local antigenic challenges and demonstrate the specificity of the mucosal memory to specific microbiota.

Microbial colonization of the gut is paired with the development of immunological memory and immune tolerance even before birth, in order to discriminate pathogenic from commensal microbiota and to ensure homeostasis. Mono-colonization of maternal germ-free mice with *B. fragilis* promotes, in 14-day pups, thymic and splenic T cell development dependent on myeloid cells migrating from the colon (Ennamorati et al., 2020). Similarly, antibiotic treatment impacts the thymic cell distribution and sensitizes the mice to colitis later in life (Ennamorati et al., 2020). Thus, the shaping of memory responses early in life reflects colonization with specific bacterial species as well as disease susceptibility, including those associated with CRC (**Figure 1B**).

## Contribution of the Microbiota to CRC

CRC refers to any cancer of the colon or rectum, regardless of their etiology. Clearly, site-specific nuances exist – including in their microbiome - but for this mini-review, the term is used broadly. The gut microbiome evolves and diversifies throughout life depending on the type of birth, diet, lifestyle, geographic factors and medications (Fiorentini et al., 2020; Dixit et al., 2021). The impact of diet and the microbiota on CRC is evidenced by data showing that mice fed a high fat diet leading to changes in their microbial communities have more disease (Yang et al., 2021). Further, the combined effects of the host response and bacteria on intestinal inflammation and CRC were illustrated in a study showing that inoculation of Rag-2-deficient mice with *H. hepaticus* induced inflammation and colonic tumors (Erdman and Poutahidis, 2017). Moreover, the decrease in the age of onset of human CRC has been associated with changes in the microbial composition in the gut, possibly due to diet or other environmental factors (Couturier-Maillard et al., 2013; Schwabe and Jobin, 2013; Allen-Vercoe and Jobin, 2014; Ray and Kidane, 2016). Based on studies like these, it has been proposed that cancer is “a failure of immune homeostasis” (Erdman and Poutahidis, 2017).

How bacteria contribute to somatic mutations associated with CRC is not well understood (Louis et al., 2014; Thomas and Jobin, 2015; Bullman et al., 2017). However, to establish causation for the microbiota in CRC, it is necessary to envisage a mechanism (Janney et al., 2020). The process of malignant transformation involves many steps including DNA damage and mutations that can be facilitated by genotoxic bacteria (Arthur et al., 2012; Arthur et al., 2014; Vizcaino and Crawford, 2015). Some organisms induce DNA damage directly while others lead to mutations through oxidative stress and changes in DNA methylation (Zouggar et al., 2020). The genetic damage induced by genotoxic bacteria and/or oxidative stress is dealt with by DNA repair pathways. Mutations or changes in the expression and function of DNA repair enzymes that respond to infections – such as AP endonuclease 1 - are associated with cancers (Wang et al., 2003; Fishel et al., 2011; Bhattacharyya et al., 2014; Sevilya et al., 2015), including CRC (Kasahara et al., 2008; Santos et al., 2014) suggesting that when DNA repair becomes compromised, somatic mutations accumulate (Taylor et al., 2003; Nooteboom et al., 2010; Tudek et al., 2010). As key genes are affected, a mutational signature emerges that favors the development of CRC. Other indirect pathways to CRC impacted by microbes and host responses include changes in local metabolites that support cell proliferation and an increase in tumor biomass (Vander Heiden et al., 2009; Datta et al., 2017; Mollace et al., 2021).

Some strains of *E. coli* produce a genotoxin that is sufficient to increase the risk of CRC (Arthur et al., 2012). Their ability to trigger inflammation may increase their oncogenic potential through effects on bacterial burden or the expression of genotoxic genes (Arthur et al., 2014). In addition, early colonization with genotoxic strains of *E. coli* decreases the protective oral tolerance response which may explain their ability to increase inflammation (Secher et al., 2015). Other species of bacteria create a dysbiosis that favors inflammation making some people more susceptible to diseases such as IBD (Chu et al., 2016; Dheer et al., 2020; Rengarajan et al., 2020; Torres et al., 2020; Dixit et al., 2021; Galipeau et al., 2021) – and by extension, increases their risk of CRC (**Figure 1B**). Inflammation in these models may be driven by the loss of bacteria that synthesize anti-inflammatory metabolites, such as short chain fatty acids (SCFAs) (Sommer et al., 2017; Zhao and Elson, 2018); or by the increase in bacteria producing pro-inflammatory molecules. For example, the bacterial production of ATP favors the induction of Th17 cells and enhances colitis (Atarashi et al., 2008; Iwase et al., 2010), while IBD has been associated with fewer SCFAs (Machiels et al., 2014) and their supplementation in animal models favorably regulates the Th17/Treg balance (Ji et al., 2019).

Other bacterial species, such as *Fusobacterium nucleatum*, have been implicated in CRC without obvious inflammation (Castellarin et al., 2012; Sears and Garrett, 2014; Garrett, 2015; Wang and Jia, 2016; Dejea et al., 2018; Yang and Jobin, 2018). While single species may be able to increase the risk of CRC, complex communities, archaea and biofilms also appear to be important (Tomkovich et al., 2019; Coker et al., 2020; Tomkovich et al., 2020). The combination of genotoxic *E. coli* and *Fusobacterium nucleatum* and enterotoxic *Bacillus fragilis* has been reported to potentiate CRC (Allen-Vercoe and Jobin, 2014; Brennan and Garrett, 2016; Fiorentini et al., 2020). While more direct evidence in humans is being sought, the data to date have led some to suggest that modifying the microbiota can protect against CRC (Dixit et al., 2021; Saeed et al., 2021).

While most of us relate to bacteria within the lumen, microorganisms with either anti- or pro-inflammatory properties have been found in the lamina propria (Sonnenberg and Artis, 2012; Dheer et al., 2020; Hosomi et al., 2020). Relevant to IBD, some microbes exist as symbionts during homeostatic conditions, but under abnormal circumstances, they assume a pathological role (Chow and Mazmanian, 2010; Devkota et al., 2012; Kamada et al., 2013). Such microbes, referred to originally as amphibionts (Blaser and Falkow, 2009) and more recently, pathobionts (Chow and Mazmanian, 2010), depend on innate lymphoid cell function (Goto et al., 2014), or Treg development (Mazmanian et al., 2008; Round and Mazmanian, 2010) linked to the production of anti-inflammatory cytokines IL-22 and IL-10 respectively, to control their pathological potential. Following a disruption in homeostasis, inflammation changes microbial gene expression to alter their colonization and virulence (Arpaia et al., 2011; Barrozo et al., 2013), sensitize host responses (Palm et al., 2014) and leaves a homeostatic “scar” that perpetuates recurrent disease (Fonseca et al., 2015). In other studies, Treg cells have been shown to both inhibit inflammation and enhance IgA (Kawamoto et al., 2014) which may explain Treg modify the microbiota and inhibit colon cancer induced by bacteria (Erdman et al., 2003). Thus, a beneficial role for homeostasis has been established.

These data reveal the direct and indirect impact that local microbes can have on host responses – as evidence of memory responses – and CRC. Should the association of microbial infection and disease extend to direct causal relationships, the argument to modify these communities will strengthen and lead to microbial manipulations for the prevention or management of disease using pre-, pro- or antibiotics (Janney et al., 2020; Dixit et al., 2021). For example, some microbial species may inhibit the growth of certain species of bacteria that express genotoxic machinery that favors DNA damage and mutations that increase the risk of cancer (Arthur et al., 2012; Pope et al., 2017).

## Consideration of Mucosal Memory Responses When Modifying the Microbiota

Targeting the microbiome alone can have some benefit in modulating diseases that lead to CRC (Sommer et al., 2017) as well as CRC itself (Dixit et al., 2021; Kim and Lim, 2021). Furthermore, it is done to enhance the efficacy of immunotherapy (Ansaldo and Belkaid, 2021). While the evidence may be incomplete, if one assumes that manipulating the microbiota will be beneficial for the prevention of at least some forms of CRC, the implications of the pre-existing immune response should be considered (**Figure 1C**). Arguments that immunological memory favors the return of the previous bacterial communities include studies showing that the composition of the microbiota rebounds after antibiotic treatment (Sommer et al., 2017). This notion is supported directly by Lindner et al (Lindner et al., 2015) who reported a persistent B cell repertoire over time in humans and mice despite infections or treatment with antibiotics. The interdependence of host-microbial interactions would be predicted by the evolutionary pressures that shape these relationships. The entire homeostatic T and B cell response that leads to the accumulation of IgA has evolved for this to occur. Clearly, memory responses that contribute to this selection could impact the effective colonization by other bacterial species – either protective or deleterious (**Figure 1D**).

Dr. Belkaid eloquently proposed the concept that a change in microbiota, associated with disease, “scars” immunological homeostasis long after the provocation has been removed (Fonseca et al., 2015). Alternatively, this “scar” may impair any attempt to re-equilibrate host responses by means of modulating the microbiota. Consequently, organisms that favor disease return or persist (Sommer et al., 2017; Lünemann et al., 2020; Galipeau et al., 2021) and trigger disease recurrence (Garrett et al., 2007). This notion is supported by a study in mice showing that anti-TNF-α used to decrease colitis changes the microbiota and attenuates the risk of developing CRC (Yang et al., 2020). Thus, treatment should break immunological barriers and allow colonization by species that favor beneficial homeostasis. This could be achieved by inhibiting “sterilizing” immunity that curtails the survival of beneficial organisms. For example, non-toxicogenic *B. fragilis* release outer membrane vesicles that induce Treg cells and prevent colitis (Mazmanian et al., 2008; Round and Mazmanian, 2010; Chu et al., 2016) while enterotoxigenic strains can favor the induction of CRC (Geis et al., 2015). As they are immunologically cross-reactive, sparing the memory responses that enable the presence of a protective strain of *B. fragilis* may be desirable. One would also want to enhance sterilizing immunity to limit colonization by “rogue” strains/species. Limiting infection is not restricted to pathogens as other species can co-exist for decades and may or may not lead to disease including cancer, e.g. *H. pylori* or genotoxic strains of *E. coli*. Therefore, altering preexisting immunity to reject unwanted species that inhabited the niche previously, perhaps through vaccination, may be needed to target pathobionts that enhance the risk of CRC and may be re-encountered in the hosts’ environment during attempts to modify the microbiome.

Mucosal antibodies do indeed, shape the microbiota (Kubinak and Round, 2016) and the molecular signature of B cells has been shown to differ in health and disease (Castro-Dopico et al., 2020). These data suggest that B cells, and likely T cells, must be “re-programmed” to allow for the optimal colonization of any transplanted microbiota and a return to homeostasis. While hard evidence is generally lacking, emerging data have led to the notion that the microbiota shape the neonatal immune system as well as following bone marrow reconstitution (Fiorenza and Turtle, 2021; Henig et al., 2021). If the microbiota and local immune response are collaborating in disease, then one would predict that totally reprograming the host response combined with modulating the microbiota would be the preferred strategy to create a new homeostatic equilibrium and a prolonged benefit. Modifying memory responses has been done by using bone marrow reconstitution to reprogram host responses in immune-mediated diseases - not just in mice but also in humans (Sellner and Rommer, 2020; Cencioni et al., 2021).

## Discussion

The vast and diverse microbial community in the intestine makes it challenging to establish a causal role for bacteria in CRC, however there are many reported associations (Castellarin et al., 2012; Sears and Garrett, 2014; Garrett, 2015; Nakatsu et al., 2015; Wang and Jia, 2016; Dejea et al., 2018; Yang and Jobin, 2018; Song and Chan, 2019; Fiorentini et al., 2020; Janney et al., 2020; Nguyen et al., 2020; Kim and Lim, 2021). The first step to establish the importance of a microbial community is to describe it and then determine if the microbe(s) transmit a phenotype. For establishing transmission – an approach that may have implications for the prevention of CRC (Kim and Lim, 2021) – one can manipulate the microbiota and determine how this impacts cancer (Pope et al., 2017). Thus, the use of fecal transplants, probiotics, prebiotics and other strategies, are credible approaches to limit the impact of microbes on CRC (Zhu et al., 2019). However, there are many variables to explore and an effective approach to treat patients by manipulating the microbiota remains to be established (Fong et al., 2020).

If immunological memory is shaping the microbiota, then mapping the antigen-binding repertoires of T and B cells would be informative to prospectively study how changes in repertoire affect the composition of the microbiota. Ideally, these assays would be performed before, during and after CRC development or treatment – with or without any manipulation of the microbiome. Introducing novel microbiota could change a substantial portion of the repertoires (Li et al., 2020) while targeted deletion of antigen-specific responses may favor colonization with new microbial species. One might also predict that a targeted strategy to neutralize a single species or pathogenic element through immunization could limit the ability of CRC-causing microbes to activate disease. As this has been challenging for *H. pylori* – a species known to cause cancer – it would be premature to propose immunizations for CRC. However, as research exposes the species and mechanisms, it could become an effective approach. As host responses to mucosal antigens are cross reactive with other microbial species, it is worth considering that some vaccines could alter the microbial balance in a deleterious, rather than a beneficial direction.

The situation is expected to be complicated by the unique properties of each microbe, host variation as well as cross-reactive host responses (Fong et al., 2020; Kabbert et al., 2020). Nonetheless, the implications are that the most successful attempt to manipulate the microbiome to prevent CRC may require parallel manipulation of the immune response, either through targeted deletions to favor colonization by beneficial microbes or the induction/maintenance of antigen-specific responses that eliminate pathogenic organisms. Many experimental tools are now in hand to address these interactions and to make targeted changes to the microbiota in CRC as effective as possible.

## Author Contributions

All authors contributed to the planning of the review. PE wrote the initial draft. LC-G formatted the manuscript and created the figure. LC-G and DR-C participated to the writing of the manuscript. JR-N, HK, and HC provided critical feedback. All authors contributed to the article and approved the submitted version.

## Funding

This publication was supported by NIH R01-AI079145 (to PE) and the Chiba University-UC San Diego Center for Mucosal Immunology, Allergy and Vaccine Development (PE, HK, HC).

## Conflict of Interest

The authors declare that the research was conducted in the absence of any commercial or financial relationships that could be construed as a potential conflict of interest.

## Publisher’s Note

All claims expressed in this article are solely those of the authors and do not necessarily represent those of their affiliated organizations, or those of the publisher, the editors and the reviewers. Any product that may be evaluated in this article, or claim that may be made by its manufacturer, is not guaranteed or endorsed by the publisher.
